# Total cucurbitacins from *Herpetospermum pedunculosum* pericarp do better than Hu-lu-su-pian (HLSP) in its safety and hepatoprotective efficacy

**DOI:** 10.3389/fphar.2024.1344983

**Published:** 2024-02-22

**Authors:** Wen-Ya Liu, Di Xu, Zi-Yun Hu, Hui-Hui Meng, Qi Zheng, Feng-Ye Wu, Xin Feng, Jun-Song Wang

**Affiliations:** ^1^ Center of Molecular Metabolism, Nanjing University of Science and Technology, Nanjing, China; ^2^ Beijing Hospital of Tibetan Medicine, China Tibetology Research Center, Beijing, China

**Keywords:** *Herpetospermum pedunculosum* pericarp, total cucurbitacins, hepatoprotective activity, NMR, median lethal dosage, no observed adverse effect level

## Abstract

The pericarp of *Herpetospermum pedunculosum* (HPP) has traditionally been used for treating jaundice and hepatitis. However, the specific hepatoprotective components and their safety/efficacy profiles remain unclear. This study aimed to characterize the total cucurbitacins (TCs) extracted from HPP and evaluate their hepatoprotective potential. As a reference, Hu-lu-su-pian (HLSP), a known hepatoprotective drug containing cucurbitacins, was used for comparison of chemical composition, effects, and safety. Molecular networking based on UHPLC-MS/MS identified cucurbitacin B, isocucurbitacin B, and cucurbitacin E as the major components in TCs, comprising 70.3%, 26.1%, and 3.6% as determined by RP-HPLC, respectively. TCs treatment significantly reversed CCl_4_-induced metabolic changes associated with liver damage in a dose-dependent manner, impacting pathways including energy metabolism, oxidative stress and phenylalanine metabolism, and showed superior efficacy to HLSP. Safety evaluation also showed that TCs were safe, with higher LD_50_ and no observable adverse effect level (NOAEL) values than HLSP. The median lethal dose (LD_50_) and NOAEL values of TCs were 36.21 and 15 mg/kg body weight (BW), respectively, while the LD_50_ of HLSP was 14 mg/kg BW. In summary, TCs extracted from HPP demonstrated promising potential as a natural hepatoprotective agent, warranting further investigation into synergistic effects of individual cucurbitacin components.

## 1 Introduction

**FIGURE 1:**
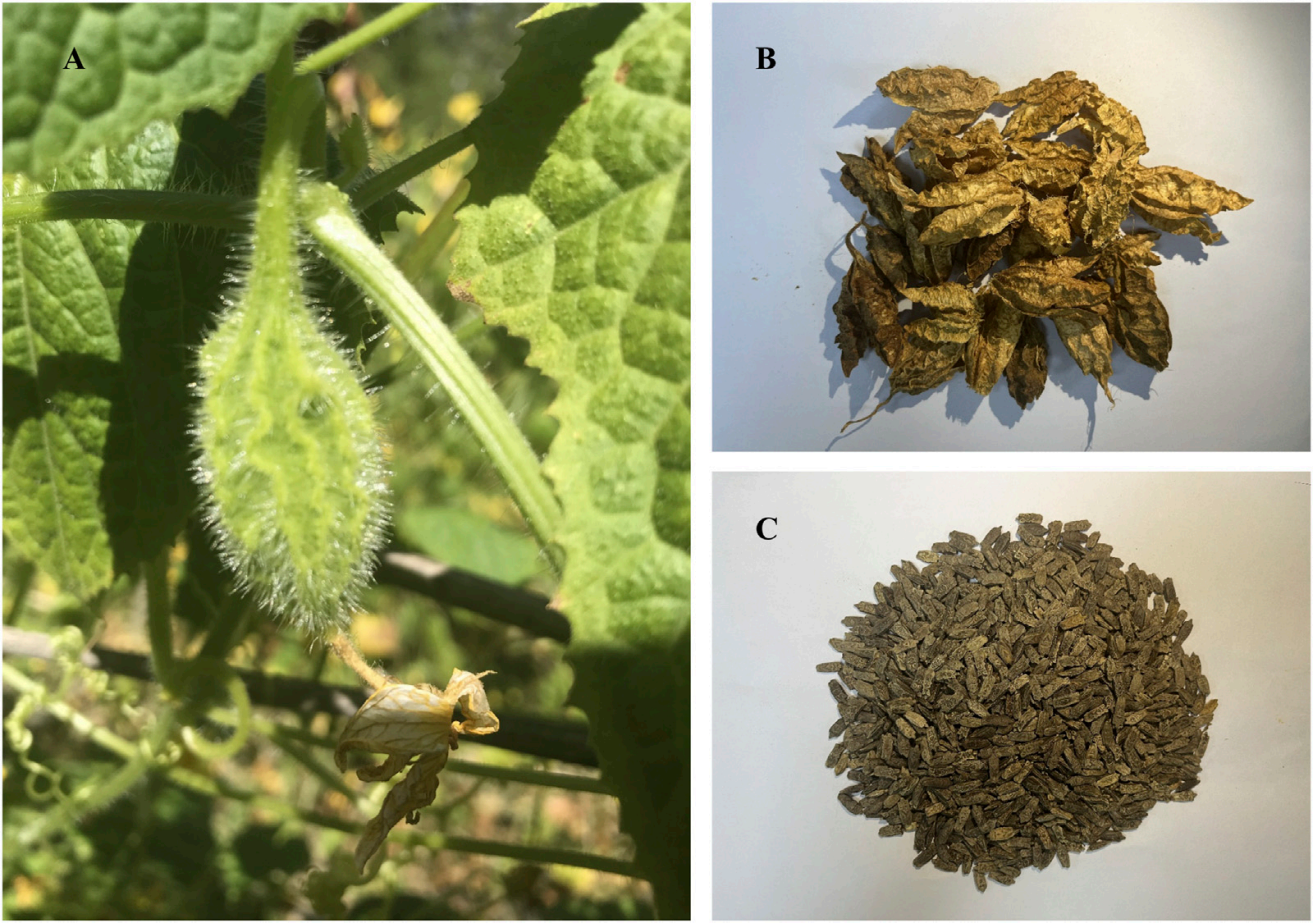
**(A)** Cucurbitaceous *Herpetospermum pedunculosum* (Ser) **(C) (B)**. Clarke and its fruit; **(B)** the dried pericarp of *Herpetospermum pedunculosum* (HPP); **(C)** the mature seed of *Herpetospermum pedunculosum* (HPS).

*Herpetospermum pedunculosum* (Ser.) C. B. Clarke (Cucurbitaceae family, Figure 1) is an annual climbing herb widely distributed in southwest China, Nepal and northeast India, growing at 2000–3,500 m altitude (Libin et al., 2003). Its dried mature seeds (HPS) known as “Se-ji-mei-duo” in Tibetan medicine (Yang, 1989), have been used for jaundice, hepatitis and dyspepsia treatment for decades. Early phytochemical studies revealed the presence of lignans (Kaouadji and Pieraccini, 1984; Dai et al., 2017), fatty acids (Zhao et al., 2009) and terpenes (Jiang et al., 2016) in HPS, conferring anti-inflammatory (Fang et al., 2007), anti-tumour (Teodor et al., 2020), anti-HBV (Gong et al., 2016), and hepatoprotective effects (Wang et al., 2014; Yu et al., 2014; Shen et al., 2015; Liu et al., 2017; Linghu et al., 2023). Our group reported the existence of series of lignans in HPS, which exhibited protection against CCl_4_-induced hepatic fibrosis (Feng et al., 2018; Li et al., 2019).

Compared to HPS (the seed, the traditional medicinal part), a significantly larger quantity of HPP is currently being discarded as industrial waste during seed processing. However, limited research has explored utilizing the HPP despite it being reported to have equivalent hepatoprotective properties (Yang, 1989) in Tibetan medicine to the seed, wasting this potential resource. Our preliminary experiments revealed that HPP is rich in total cucurbitacins (TCs), which are tetracyclic triterpenoids differing in functional groups and ring saturation (Chen et al., 2005; Wang et al., 2017). Cucurbitacins are characteristic components of Cucurbitaceae plants, attracting attention for diverse activities including anti-inflammatory (Peters et al., 1997; Bernard and Olayinka, 2010; Marzouk et al., 2013; Nagarani et al., 2014; Silvestre et al., 2022), anti-tumor (Jayaprakasam et al., 2003; Molavi et al., 2008; Lee et al., 2010), and hepatoprotection (Bartalis, 2005; Asadi-Samani et al., 2015; Arjaibi et al., 2017) over decades. However, there are few studies on the chemical composition of TCs from HPP and whether they have hepatoprotective effects.

Hu-lu-su-pian tablets (HLSP, approval number: Z43021002) have been utilized in the medicinal market since the 1980s for the treatment of hepatitis and primary hepatocellular carcinoma (Hu et al., 1982; Mei et al., 2021; Kanani and Pandya, 2022). However, the clinical application of HLSP has been limited due to adverse reactions such as diarrhea, dizziness, and nausea following drug administration. HLSP is derived from the fruit stalk of *C. melo* L (Cucurbitaceae family) and has a historical usage in Chinese medicine for treating liver diseases. The fruit stalk of *Cucumis melo* was first documented in the ancient Chinese Pharmacy monograph, Shen Nong Ben Cao Jing (Wu, 2016), compiled around 200 BC. Subsequently, in the influential Materia Medica compiled by Li Shizhen during the 16th century AD, its efficacy in treating jaundice was emphasized (Li, 1975). While *C. melo* itself is an edible plant, the fruit stalk concentrates cucurbitacins as a chemical defense mechanism. Similarly, HPP also biosynthesizes cucurbitacins for defense purposes. However, cucurbitacins exhibit significant chemical variations and proportions among different Cucurbitaceae plants, which can result in diverse biological effects in terms of medicinal value, toxicity, antioxidant properties, anti-inflammatory properties, and antibacterial properties. By comparing the cucurbitacin profiles in HPP and the HLSP product, it becomes possible to evaluate HPP as a potential alternative medicinal source to the established HLSP. Therefore, a comprehensive evaluation of the chemical composition, proportion, safety, and effectiveness of TCs and HLSP is necessary.

This study aims to compare the hepatoprotective efficacy and safety of total cucurbitacins (TCs) extracted from fruit pericarp (HPP) of *H. pedunculosum* to HLSP. While similar active components may confer comparable therapeutic effects, variations in chemical profiles can impact safety and efficacy. We performed UHPLC-MS/molecular networking to qualitatively analyze and compare TCs in HPP and HLSP. Metabolic profiling and mice studies were then used to comprehensively evaluate the hepatoprotective effects and safety of TCs from HPP *versus* HLSP. Comparing HPP to the more toxic HLSP may identify potential candidates from HPP for safer hepatoprotection, advancing development and utilization of this understudied plant resource.

## 2 Materials and methods

### 2.1 Reagents and materials

Methanol and acetonitrile of HPLC grade were bought from Tedia Company (Fairfield, United States). 3-(trimethylsilyl) propionic-2, 2, 3, 3-d_4_ acid sodium salt (TSP) and Deuterium oxide (D_2_O, 99.9%) were purchased from Sigma-Aldrich (St. Louis, MO, United States). Deionized water was ordered from Watsons (Watsons, Hong Kong, China). The three standards, including Cucurbitacin B (CuB), Isocucurbitacin B (IsocuB), and Cucurbitacin E (CuE) were laboratory-made and identified by^1^H and ^13^C NMR. Hu-lu-su-pian (HLSP, the main ingredients are CuB and CuE, 0.1 mg/tablet) was used as a positive drug and was obtained from Hunan Dinuo Pharmaceutical Co., Ltd. (Changsha, China). The Nanjing Jiancheng Bioengineering Institute (Nanjing, China) supplied the assay kits for the enzymes aspartate aminotransferase (AST) and alanine aminotransferase (ALT). HPP were provided by Beijing Tibetology Research Center (Linzhi City, Tibet Autonomous Region, China). Other reagents were all analytically pure.

### 2.2 Preparation of TCs

5 kg HPP was crushed to 30 mesh with a multifunctional crusher, and then extracted twice with ethanol (1:10, w/v) under reflux for 2 h each time. The two filtrates were collected and concentrated under reduced pressure to obtain 390 g of an ethanol extract. The extracts were extracted using petroleum ether and ethyl acetate after being suspended in water. The extract (48.5 g) of ethyl acetate was successively eluted with petroleum ether-acetone (8:2, 7:3, 6:4, 5:5, 4:6, v/v) on a silica gel column (50 cm × 8 cm i.d.), and the thin fractions were checked by thin layer chromatography (TLC). Fractions 11-14 were collected, concentrated under reduced pressure, and fully dried in a vacuum drying oven to obtain 3.42 g of TCs.

### 2.3 Qualitative profiling of TCs and HLSP by UHPLC-QTOF-MS/MS

#### 2.3.1 UHPLC-Q-TOF MS analysis

An UHPLC-Q-TOF MS system comprised of an AB SCIEX Triple-TOF 5600^+^ mass spectrometer (MA, United States) and a SCIEX Exion ultra-high performance liquid chromatography (UHPLC) system was used to conduct the LC-MS analysis (Hu et al., 2023). LC separation was performed using a Phenomenex Kinetex^®^ Biphenyl C18 column (100 × 2.1 mm i.d., 2.6 m; Torrance, CA, United States) with a flow rate of 0.4 mL/min at 40°C. As mobile phases, formic acid in water (solvent A, 0.1%, v/v) and acetonitrile (solvent B) were employed. The procedure for a gradient elution was as follows: 0–1 min, 1% B; 1–10 min, 1–99 %B; 10–13 min, 99% B; 13–14 min, 99%–1% B; 14–17 min, 1% B. the inject volume was 2 μL.

Information-dependent acquisition (IDA) was used to acquire the MS data, which included a TOF MS scan and an intensity-dependent TOF MS/MS scan with high sensitivity mode selected. In the TOF MS-IDA-MS/MS acquisition, the TOF MS spectral mass scanning range was 50–1,000 m/z, the accumulation time was set at 0.10 s/spectrum, and the product ion scanning mass range was 40–1,000 m/z, with the accumulation time being 0.05 s/spectrum. The following settings were made for the IDA mode: maximum candidate ions, 10; mass tolerance, 50 mDa; declustering potential (DP), 80 V (ESI^+^)/−80 V (ESI^−^); collision energy (CE), 35 V ± 15 V (+)/−35 V ± 15 V (−); intensity threshold, 100 cps; with the dynamic background subtraction (DBS) function on. The following detailed ESI settings were made: source temperature was set to 550°C, nebulizing gas (GS1) was set to 55 psi, auxiliary gas (GS2) was set to 55 psi, curtain gas was set to 35 psi, and spray voltage was set to 5,500 V (+)/−4500 V (−). Calibration was carried out using an external calibration reference to ensure mass accuracy before injection.

#### 2.3.2 Molecular networking

Molecular networks were established by the online workflow at Global Natural Products Social Molecular Networking (GNPS) platform (http://gnps.ucsd.edu).The raw MS data (.wiff format) were obtained by SCIEX Analyst TF 1.8.1 Software (version 1.8.1, MA, United States), they were first converted into.mzML files by ProteoWizard-MS Convert (version 3.0, Proteowizard Software Foundation, CA, United States) and then uploaded on the GNPS Web platform (Wang et al., 2016; Quinn et al., 2017; Ramos et al., 2019). The following parameters were used for the development of molecular networks: both the precursor and fragment ion mass tolerance were of 0.02 Da, molecular networking was constructed using 10 minimum matched fragment ions and a minimum cosine score of 0.8, the other parameters were default values. The MS/MS molecular network is accessible at the GNPS Web site with the following link: https://gnps.ucsd.edu/ProteoSAFe/status.jsp?task=78587ed5c58f4cfbac4923ede9b305a8. Data were visualized using Cytoscape 3.6.0 software (https://cytoscape.org/).

Formula Finder and the structural elucidation tool inside SCIEX OS Software (version 2.0, MA, United States) were used to further assess the MS and MS/MS data for characteristics of interest.

### 2.4 Quantitative analysis of TCs and HLSP by HPLC

#### 2.4.1 Chromatographic conditions

The TCs analysis (Chanda et al., 2020) was carried out using a Shimadu LC-20AT series HPLC system (Shimadu, Tokyo, Japan) with an autosampler and an InertSustain C18 column (250 × 4.6 mm, 5 μm). A mobile phase consisting of 50% acetonitrile and water was used on an isocratic elution procedure at a flow rate of 1 mL/min over 30 min, the column temperature was 30°C, and 230 nm was selected as detection wavelength.

#### 2.4.2 Standard curve creation

The quantitative analysis of the three cucurbitacins was performed by the use of an external standard method (Scherer et al., 2012), and a mixed standard solution containing CuB (2.4 mg), IsocuB (0.64 mg) and CuE (0.32 mg) was prepared in a 1 mL volumetric flask. Use the double dilution method to serially dilute 4 times, so that the concentration of CuB is 0.15, 0.3, 0.6, 1.2, 2.4 mg/mL, and the concentration of IsocuB is 0.04, 0.08, 0.16, 0.32, 0.64 mg/mL, the concentrations of CuE were 0.02, 0.04, 0.08, 0.16, 0.32 mg/mL. A 10 μL aliquot of each solution was injected and analyzed three times using HPLC, and the standard curves were obtained by plotting peak area *versus* concentration.

#### 2.4.3 Content determination

Accurately weigh 10 mg of TCs and prepare a 1 mg/mL test solution with methanol in a 10 mL volumetric flask. Take 10 tablets of HLSP, grind in a mortar, 5 mL of methanol was added, then ultrasonic extraction was performed twice for 30 min, the two extract solutions were combined and transferred into a 10 mL volumetric flask and diluted with methanol to volume. After filtration through a 0.22 μm microporous membrane, it was used as another test solution. According to the chromatographic conditions of 2.4.1, an injection volume of 10 μL sample solution was performed, the contents of three components were determined based on the corresponding peak area by external standard method.

### 2.5 Animal experiments

ICR mice (20.0 ± 2 g) were obtained from Qinglongshan Animal Breeding ground in Nanjing. All animals were kept in a specified pathogen-free (SPF) laboratory with unrestricted access to food and water at a temperature of 25°C, with a humidity of 55% ± 5%, and a 12-h light/dark cycle. The animals used in this study have been handled in accordance with the National Institutes of Health (NIH) guidelines, and the experimental protocol has been approved by the Animal Care and Use Committee of Nanjing University of Science and Technology.

#### 2.5.1 Safety assessment of TCs—LD_50_ and NOAEL

We employed a modified “up and down” method (Abal et al., 2017) with four dose levels to assess the safety of TCs and determine the LD_50_ and NOAEL. Female ICR mice weighing 20.0 ± 2 g were fasted overnight prior to administration of a single dose of TCs (20 mL/kg BW, suspended in 2% Tween-80 physiological saline) by gavage the following morning. After that, the animals were kept in metabolic cages with free access to food and water for the following 2 weeks. The first, second, third, and fourth dose levels were tested on groups of three, five, seven, and nine mice, respectively, with a starting dose of 80 mg/kg BW. Doses were decreased if more than 50% of the mice died or increased if less than 50% of the mice died. Detailed observations and recordings of symptoms, including apathy, piloerection, shortness of breath, slight convulsion, semi-closed eyes, and survival time, were recorded after gavage administration for each mouse.

#### 2.5.2 Metabolomics study

After 7 days of acclimation in the laboratory environment, male ICR mice were randomly divided into 6 groups (10 mice in each group): normal control (NC) group, CCl_4_ group, HLSP group, the low dose of TCs-treated (LD) group, the medium dose of TCs-treated (MD) group and the high dose of TCs-treated (HD) group. The LD, MD and HD groups were gavaged with TCs (suspended in 0.5% CMC-Na solution) at 0.1, 0.2, 0.4 mg/kg BW, respectively. Mice in the HLSP group were fed with HLSP tablets (equivalent to a MD of TCs), and the 0.5% CMC-Na solution was administered in the same amount to the NC and CCl_4_ groups. In addition, the body weight of each mouse was recorded every morning at 9 a.m. All groups were treated once a day for consecutive 7 days (Yang et al., 2015). After 2 h of administration on the 7th day, except for the NC group, mice in the other groups were intraperitoneally injected with 0.3% CCl_4_ olive oil (10 mL/kg BW), while the NC group was given an equal volume of olive oil. Mice in each group were fasted for 24 h, and then were anesthetized with isoflurane controlled by a small animal anesthesia machine (medical supplies andservices INT. LTD. Keighley, UK). The orbital blood was immediately collected for serum biochemical analysis and ^1^H NMR analysis. Each mouse was then sacrificed, the liver was collected for histopathological examination and ^1^H NMR analysis, and the relative liver weight was calculated (relative liver weight (%) = liver weight/body weight × 100). Blood samples were centrifuged (3,000 rpm, 10 min, 4°C) to obtain serum samples and stored at −80°C until biochemical analysis.

### 2.6 Histopathological evaluation and biochemical analysis

For histopathology, liver tissues were quickly removed, and then fixed with 10% formalin to prepare paraffin sections with 5 μm sections for H&E staining, and the remainder were stored at −80°C for other analyses.

For serum biochemical analysis, ALT and AST levels were assessed using a commercial kit in accordance with the manufacturer’s recommendations.

### 2.7 Liver and serum preparation for ^1^H NMR analysis

Liver tissue sample preparation for ^1^H NMR analysis (Ruan et al., 2018): Frozen liver tissue samples were crushed in a mortar and pestle in the presence of liquid nitrogen and immediately weighed, then homogenized (5 mL/g) using pre-cooled acetonitrile-water (50:50, v/v). The homogenate was centrifuged for 10 min at 16,000 g at 4°C, and the supernatant was then transferred to a centrifuge tube. With the removal of acetonitrile under a nitrogen blower, it was then lyophilized and kept at −80°C for future use.

Serum sample preparation for ^1^H NMR analysis (Fu et al., 2019): Slowly thawing serum samples on ice was followed by the addition of twice as much methanol, the mixture was vortexed and left to stand at −20°C for 20 min. The supernatant was evaporated with a nitrogen blower to remove methanol following centrifugation at 14,000 g for 15 min at 4°C. It was then freeze-dried and kept at −80°C for future use.

The dried liver and serum extracts were added with a new mixture of 600 μL99.8% D_2_O phosphate buffer (0.2 M, Na_2_HPO_4_ and 0.2 M NaH_2_PO_4_, pH 7.0) containing 0.05% (w/v) sodium 3-(trimethylsilyl) propionate-2,2,3,3-d_4_ (TSP). After vortex and 14,000 g centrifugation for 10 min, the supernatant was transferred to the 5 mm NMR tube for the ^1^H NMR analysis.

### 2.8 ^1^H NMR recording

We acquired ^1^H NMR spectra of liver and serum samples using a Bruker Avance 500 MHz spectrometer (Bruker GmbH, Karlsruhe, Germany). The pulse sequence was edited with a lateral (perpendicular to the main magnetic field direction) relaxation-edited Call-Purcell-Meiboom-Gil (CPMG) sequence (90 (τ-180-τ) n-acquisition) and a spin echo delay of 10 ms (2 nτ) in order to suppress residual macromolecular protein signal in the sample. The number of scans was 32 (32 K data points) and the spectral width was 20 ppm. The spectra were Fourier transformed after multiplying the free induction decay (FID) curve by an exponential weighting function (corresponding to a 0.5 Hz linewidth).

### 2.9 Data preprocessing and multivariate statistical analysis of ^1^H NMR data

Using the software Topspin 3.0 from Bruker BioSpin, all NMR spectra were manually pre-processed for baseline, phase, and TSP zero (0.0 ppm). MestReNova (version 6.1.0, Mestrelab Research SL) was used to convert the files to ASCII format, which was then imported into “R” (http://cran.r-project.org/) for multivariate statistical analysis. To minimize the data dimension, segmental integration is carried out in the chemical potential range of 0.5 ppm–9 ppm using an adaptive binning approach with an average bin width of 0.015 ppm. Between 4.47 and 5.50 ppm, the remaining signals of water and its affected regions were eliminated. Finally, the data were subjected to Pareto scaling (Karaman, 2017) and probability quotient normalization (Dieterle et al., 2006) to eliminate systematic differences between sample concentrations.

Orthogonal signal correction-partial least squares discriminant analysis (OSC-PLSDA) was then performed on the processed data, revealing differential metabolic changes in serum and liver tissue. The orthogonal signal correction (OSC) filter is applied to remove uninteresting variations, such as systematic variation, from spectral data before performing PLS-DA. To evaluate the fitting ability and predictive ability of the established OPLS-DA model, repeated two-fold cross-validation (2CV) and permutation test (*n* = 200) were performed. *R* ^2^ (total explained variance) and Q^2^ (model predictability) values were used to assess the model’s quality. Differences and clusters between groups were displayed using score plots, and metabolites that changed between groups were displayed using colored loading plots.

### 2.10 Metabolites identification and univariate analysis

Metabolites of liver tissue and serum were identified using the commercial software Chenomx NMR Suite v.8.1 (Chenomx, Edmonton, Canada) and statistical total correlation spectroscopy (STOCSY) techniques, and then by querying public metabolite databases such as the Human Metabolite Database (HMDB, http://www.hmdb.ca) and the Madison-Qingdao Metabolomics Database (MMCD, http://mmcd.nmrfam.wisc.edu).The preliminary identification of metabolites was further confirmed by using 2 dimensional (2D) heteronuclear single quantum correlation (HSQC) and a ^1^H–^1^H total correlation (TOCSY) NMR spectroscopy.

For univariate analysis, a parametric test (*t*-test) and a non-parametric statistical test (Wilcoxon signed rank test) were employed to validate key increased or decreased metabolites across groups using “R”. The fold change (FC) values of metabolites adjusted by the Benjamin-Hochberg modification approach and their correlated *p* values were calculated and exhibited as colored tables. The significance threshold for all tests was set at *p* < 0.05.

### 2.11 Statistical analysis

All data are expressed as mean ± SD, and statistical analysis was performed using GraphPad Prism 8.0 software (GraphPad Software, CA, United States).

## 3 Results

### 3.1 Characterization of the major cucurbitacins in TCs and HLSP by molecular networking

**FIGURE 2:**
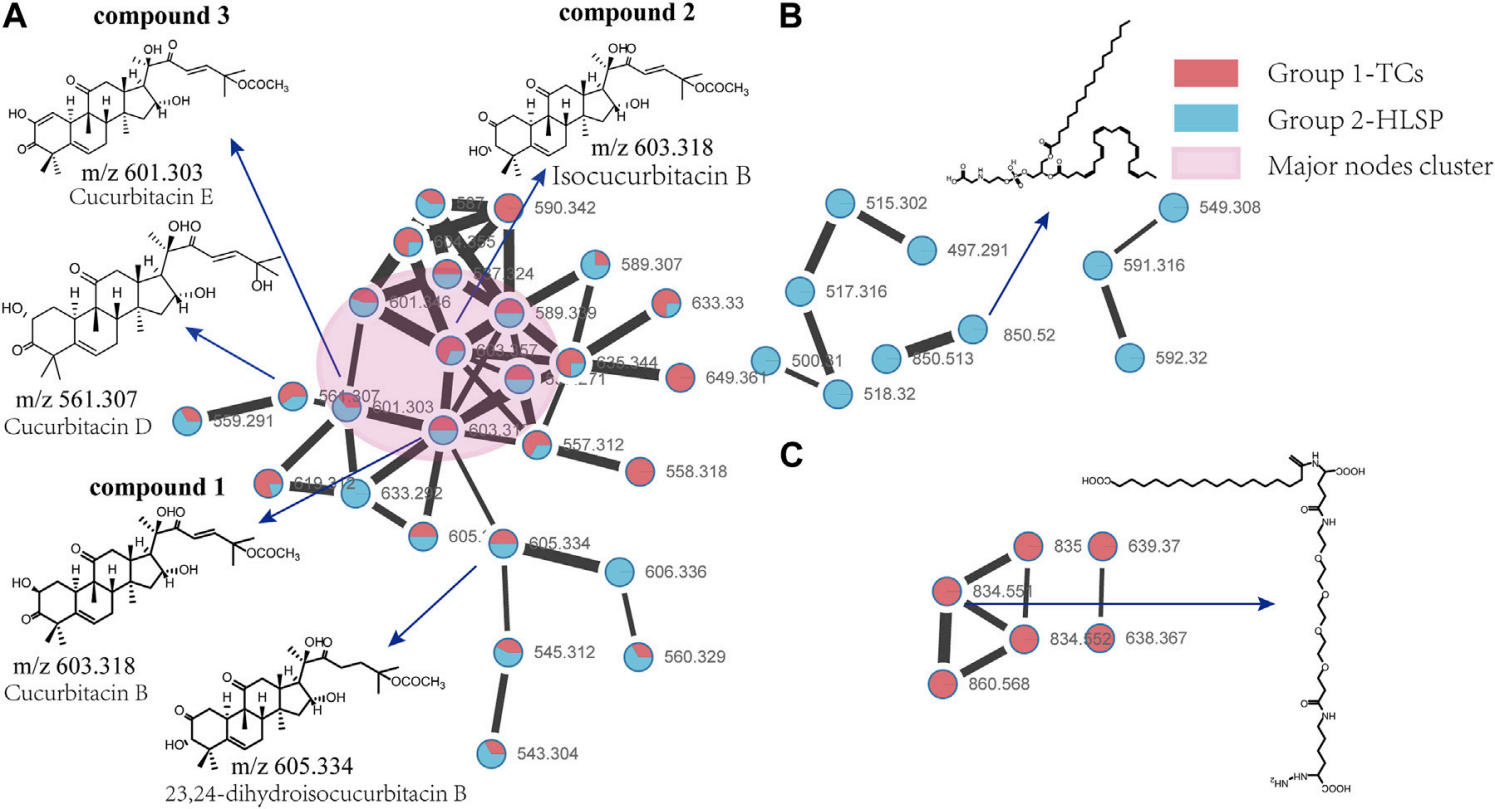
Molecular Network of TCs and HLSP obtained using GNPS platform and visualized with cytoscape 3.6.0 software. **(A)** Shared nodes with TCs and HLSP; **(B)** Blue and **(C)** red nodes represent the unique compounds of HLSP and TCs, respectively. The pink part was the major nodes cluster shared with TCs and HLSP.

Molecular networking is a powerful complement to screening and identification of undescribed compounds from natural products (Yang et al., 2013). The molecules exhibiting similar fragmentation patterns were clustered together while the molecules with dissimilar MS/MS spectra were displayed as independent nodes (Wang et al., 2016). The visualized molecular networking of TCs and HLSP using Cytoscape 3.6.0 software is presented in Figure 2. Each node represents a compound, inside which the molecular mass of parent ion of the compound is indicated. The nodes of compounds are linked by lines indicating structural similarity, with thicker lines representing greater similarity between the structures. The red and blue node represents TCs and HLSP, respectively. The Pink area represents major cucurbitacin clusters. Clusters that formed by red nodes and blue nodes represent the unique compounds of TCs (Figure 2B) and HLSP (Figure 2C), respectively. Compounds 1 and 2 showed identical [M + FA-H]^−^ ions at m/z 603.3 in negative ion mode, suggesting that they were isomers. Furthermore, their MS/MS spectra exhibited the typical daughter ions at m/z 497.3, corresponding to the loss of CH_3_COOH (60 Da). In addition, the other fragment ions were nearly alike except for some disparities in relative abundance (Supplementary Figures S1, S2). This revealed that they were two configurational isomers. Thus, Compounds 1 and 2 were therefore tentatively identified as cucurbitacin B and isocucurbitacin B by comparing their retention times and MS/MS spectra with those of authentic standards.

Compound 3 showed a [M + FA−H]^−^ ion at m/z 601.3, as well as the typical daughter ion at m/z 495.3 which were 2 Da less than compound 1 and 2 (Supplementary Figures S1, S2), indicating that the core structure of compound 3 contained one additional double bond compared to Compound 1 and 2. Therefore, by comparing its retention time and MS/MS spectra with those of an authentic standard, compound 3 was tentatively identified as cucurbitacin E. The retention time, molecular formula, mass errors, MS/MS fragment ions, and detected monoisotopic masses in the four-decimal format provided by QTOF-MS for the major cucurbitacins and the other nine derivatives are summarized in Table 1. Other untargeted compounds (with an MS^2^ spectrum) were also annotated by matches to the GNPS spectral libraries (data not shown).

**TABLE 1:** Identification of cucurbitacins in TCs and HLSP by UHPLC–QTOF–MS-MS.

	Rt		Addut ions (measured)		MassError (ppm)	MS/MS fragments		
No.	(min)	Formula	[M + Na]+	/[M + FA-H]^-^	ESI(+/−)	ESI(+)	ESI(−)	Identification
1	6.612	C_32_H_46_O_8_	581.3075	603.3178	−1.7/0.6	581.3054, 521.2845	603.3147, 557.3108, 539.3020, 497.2886, 411.2180, 301.1435, 59.0138	Cucurbitacin B
2	6.737	C_32_H_46_O_8_	581.3069	603.3173	−2.8/-0.3	581.3049, 521.2839	603.3173, 557.3102, 539.3011, 497.2811, 411.2167, 385.2013, 59.0133	Isocucurbitacin B
3	6.966	C_32_H_44_O_8_	579.2914	601.302	−2.5/0.3	579.2906, 519.2691	601.2986, 555.2944, 537.2854, 495.2737, 409.2015, 299.1275, 59.0134	Cucurbitacin E
4	6.027	C_3_0H_42_O_7_	537.2816	559.2895	−1.2/-3.2	537.2790, 281.0507	559.2888, 513.2820, 495.2712, 477.2561, 462.2493, 163.0797, 44.9983	Cucurbitacin I
5	5.780	C_30_H_44_O_7_	539.2971	561.3059	−1.5/-1.8	539.2934	561.3013, 515.3010, 497.2902, 479.2852, 455.2763, 385.2206, 301.1819, 165.0929	Isocucurbitacin D
6	5.857	C_30_H_44_O_7_	539.2954	561.3081	−4.6/2.1	539.2938	561.3081, 515.2976, 501.3167, 255.1703, 165.0916, 59.0140	3-epi-isocucurbitacin D
7	6.704	C_32_H_48_O_8_	583.3209	605.3302	−5.6/-4.8	583.3166, 523.2981	605.3283, 559.3268, 541.3158, 499.3041, 481.2957, 341.2121, 301.1812, 165.0922, 59.0142	23,24-dihydrocucurbitacin B
8	6.850	C_32_H_48_O_8_	583.3214	605.3340	−4.7/1.4	583.3206, 523.2996	605.3287, 559.3288, 541.3199, 499.3038, 481.2929, 301.1807, 165.0926, 59.0134	23,24-dihydroisocucurbitacin B
9	7.079	C_32_H_46_O_8_	581.3061	603.3180	−4.2/0.9	581.3030, 521.2815	603.3123, 557.3108, 539.2982, 497.2890, 479.2789, 163.0765, 59.0141	3-epi-isocucurbitacin B
10	5.695	C_30_H_46_O_7_	541.3111	563.3202	−4.6/-2.4	541.3111, 523.2956	563.3121, 517.3149, 499.3057, 385.2394, 165.0911, 137.0948	23,24-dihydrocucurbitacin D
11	5.703	C_30_H_44_O_7_	539.2958	561.3050	−3.9/-3.5	539.2961	561.3033, 515.2968, 497.2845, 479.2748, 439.2458, 165.0935, 59.0129	Cucurbitacin D
12	5.765	C_30_H_46_O_7_	541.3098	563.3209	−6.2/-4.2	541.3101, 523.3070	563.3121, 517.3149, 499.3048, 457.3000, 385.2373, 165.0932, 59.0133	dihydro-epi-iso-cucurbitacin D

### 3.2 Contents of CuB, IsocuB and CuE in TCs and HLSP

**FIGURE 3:**
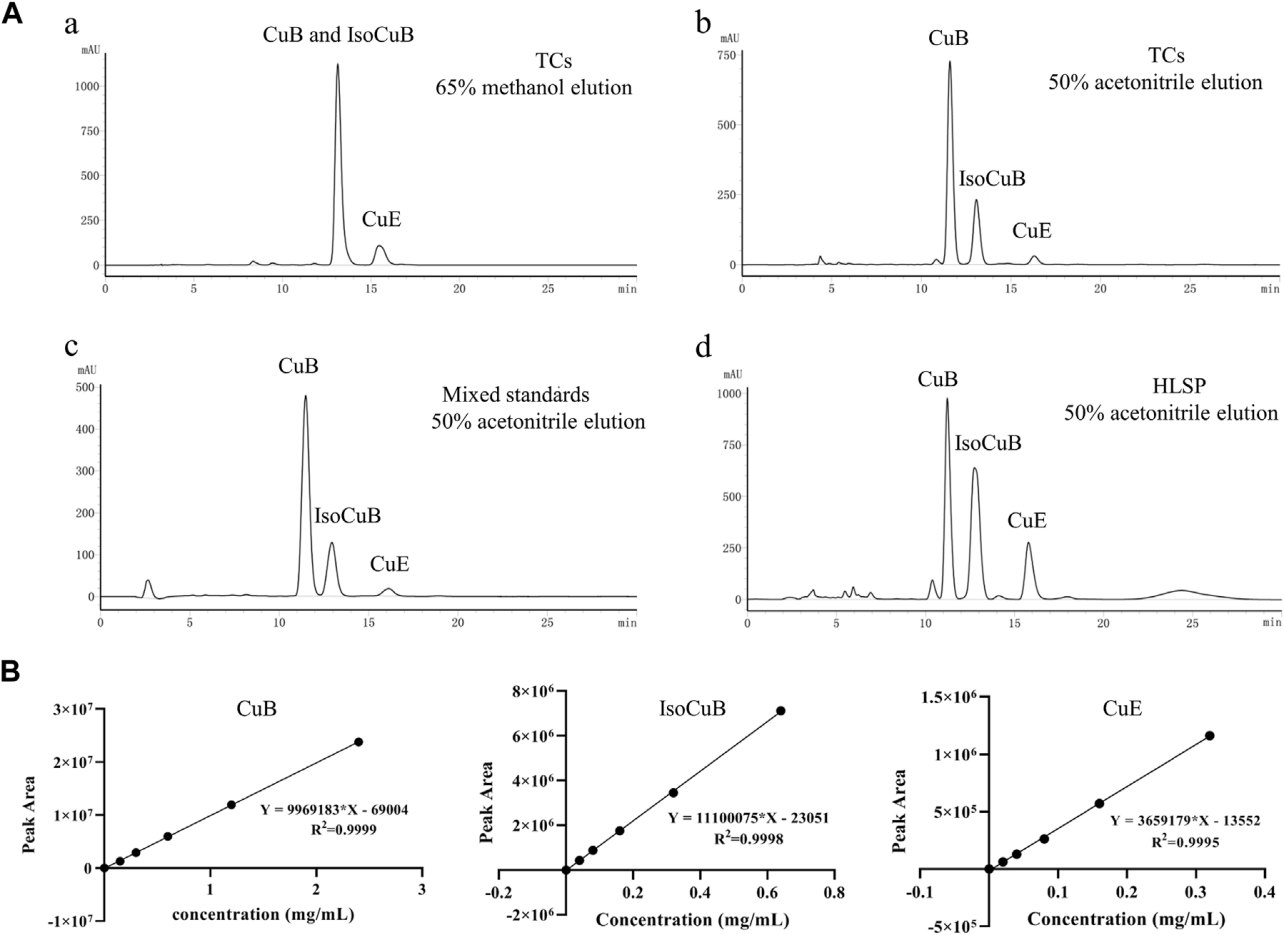
**(A)** HPLC chromatograms of TCs and HLSP, (a) TCs eluted with 65% methanol; (b) TCs eluted with 50% acetonitrile; (c) mixed standards eluted with 50% acetonitrile; (d) HLSP eluted with 50% acetonitrile. **(B)** Standard curves for CuB, IsocuB and CuE.

In order to further investigate the amount of each component in TCs, three primary components were quantified by HPLC technique. CuB and IsocuB could not be completely separated by 65% methanol (Figure 3Aa), whereas the two overlapping isomers could be completely separated by 50% acetonitrile (Figures 3Bb, c, d) with a resolution R > 1.5, therefor, an isocratic elution with 50% acetonitrile was used to determine CuB, IsocuB, and CuE in TCs and HLSP (Figure 3B). The contents of CuB, IsocuB, and CuE were respectively 70.3% ± 0.4%, 26.1% ± 0.2%, and 3.6% ± 0.3% in TCs and 41.0% ± 1.1%, 42.8% ± 0.7% and 16.2% ± 0.6% in HLSP, as shown in Table 2.

**TABLE 2:** Content of CuB, IsocuB and CuE in TCs and HLSP (n = 3, mean ± SD).

No.	Name*	TCs			HLSP		
		tR (min)	Peak area (×10^6^)	Content (%)	tR (min)	Peak area (×10^6^)	Content (%)
1	CuB	11.5 ± 0.1	15.8 ± 0.2	70.3 ± 0.4	11.5 ± 0.3	21.7 ± 0.9	41.0 ± 1.1
2	IsocuB	13.0 ± 0.1	5.9 ± 0.06	26.1 ± 0.2	12.9 ± 0.2	22.6 ± 0.6	42.8 ± 0.7
3	CuE	16.1 ± 0.2	8.2 ± 0.05	3.6 ± 0.3	16.1 ± 0.3	8.6 ± 0.2	16.2 ± 0.6

### 3.3 Safety assessment of TCs

**FIGURE 4:**
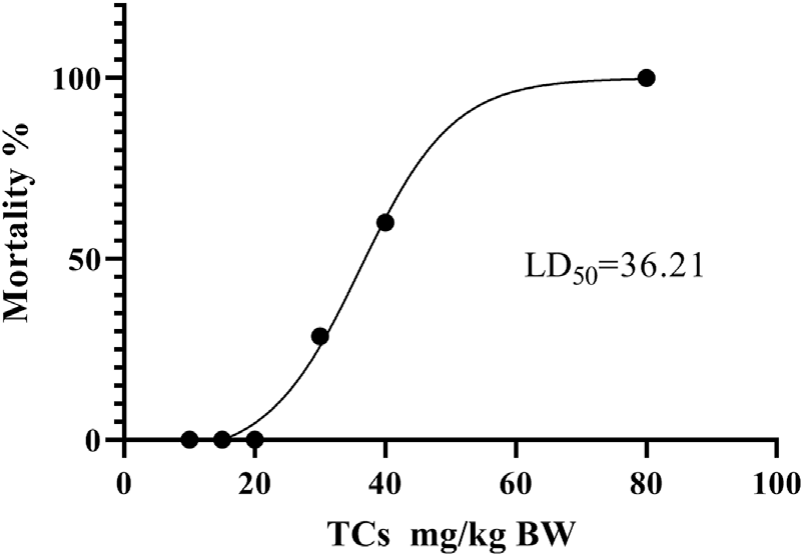
Dose-response mortality curve of oral TCs in mice. Percentage mortality *versus* concentration of the TCs. The LD_50_ value is indicated.

LD_50_ and NOAEL values are two metrics commonly used to assess the safety of drugs (Vilariño et al., 2018). Following gavage administration of TCs, all mice died within 5 hours at a dosage of 80 mg/kg. Therefore, the second level dosage was reduced to 40 mg/kg, leading to the death of three out of five mice. At the third level, a dosage of 20 mg/kg resulted in no deaths, prompting a further reduction to 10 mg/kg for the fourth level. None of the nine mice who received this dose died. An additional dose level of 30 mg/kg was introduced between the second and third levels, resulting in the death of two out of seven mice. Lastly, nine mice were given a dosage of 15 mg/kg, with no deaths observed (Table 3). Symptoms of apathy and piloerection were observed within 30 min across all groups, except at 15 and 10 mg/kg. Convulsions increased in frequency over time, occurring every 15 s. Semi-closed eyes were observed an hour before death. At a dosage of 20 mg/kg, 1 mouse showed signs of apathy and another had a minor piloerection. At 15 and 10 mg/kg, none of the mice died and there were no side effects over the following 2 weeks (Table 4). Based on these findings, the oral NOAEL for mice was established at 15 mg/kg, and this modified four-level “up and down” procedure revealed dose-dependent mortality. The results, presented as a percentage of mouse deaths in relation to TCs dosage, are shown in Figure 4. Nonlinear regression fitting software was used to calculate the oral LD_50_ of TCs to be 36.21 mg/kg (GraphPad Prism, version 8.0, GraphPad Software, CA, United States).

**TABLE 3:** Mortality induced by gavage administration of TCs to mice and survival times corresponding with each treatment.

Does (mg/kg)	Mortality	Survival Times(h)
80	3/3	2.53, 3.67, 4.9
40	3/5	2.85, 3.75, 5.1
30	2/7	14.69,14.69–20.5
20	0/7	>336
15	0/9	>336
10	0/9	>336

**TABLE 4:** Symptoms registered after TCs administration. The ratio between mice with the symptom *versus* the total mice treated.

Symptoms	TCs Dose (mg/kg)					
	80	40	30	20	15	10
Apathy	3/3	5/5	7/7	2/9	0/9	0/9
Piloerection	3/3	3/5	5/7	1/9	0/9	0/9
Shortness of breath	1/3	1/5	0/7	0/9	0/9	0/9
Slight convulsion	3/3	0/5	2/7	0/9	0/9	0/9
Semi-closed eye	3/3	4/5	4/7	0/9	0/9	0/9

### 3.4 Effects of TCs on body weight, relative liver weight, serum AST and ALT activities

**FIGURE 5:**
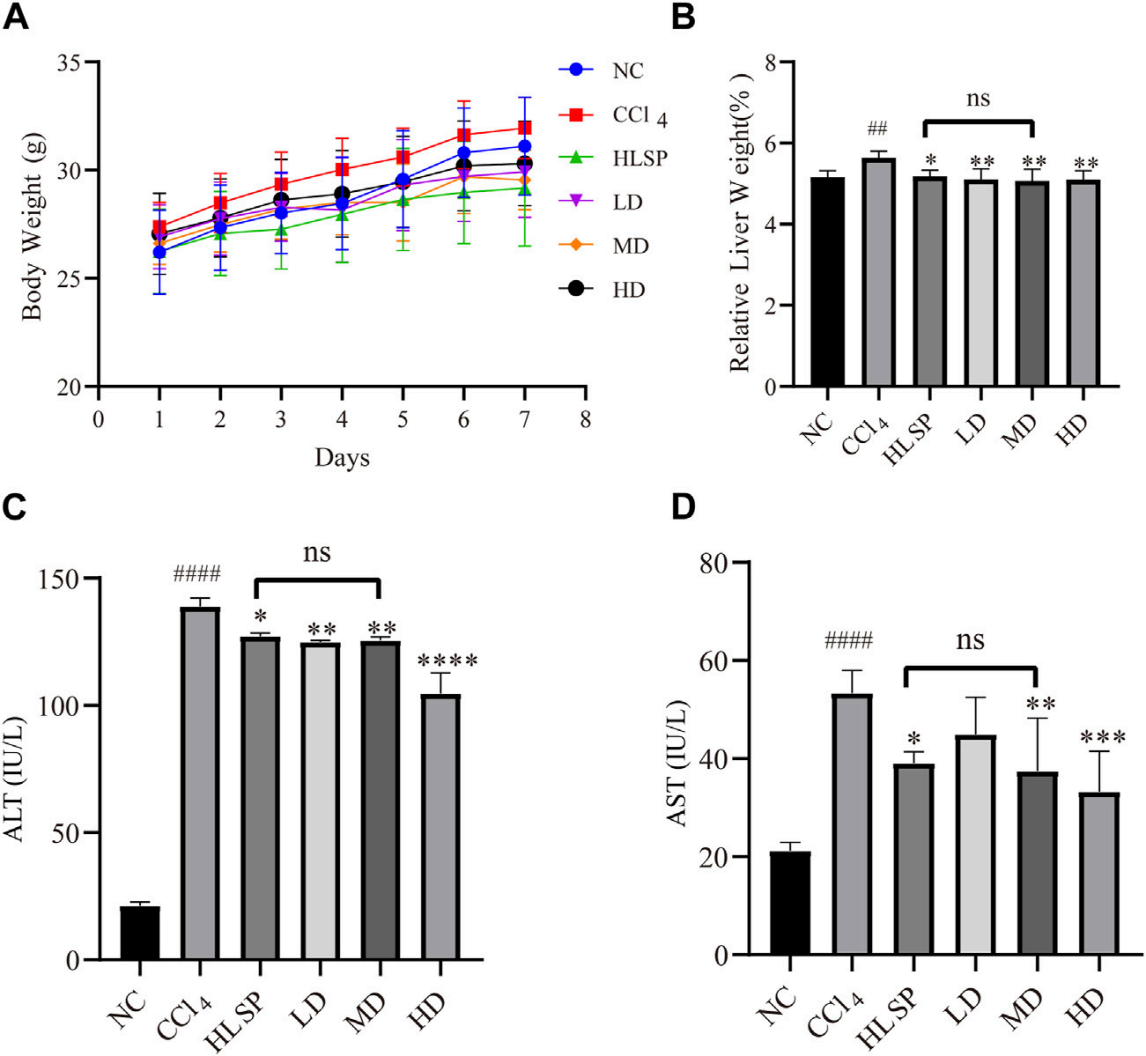
Effects of TCs on body weight and relative liver weight, serum AST and ALT activities inCCl_4_ -induced mice. **(A)** Body weight changes, **(B)** serum biochemical levels (*n* = 5) relative to liver weight (*n* = 10), ALT **(C)** and AST **(D)**. Data are expressed as mean ± SD. Compared with NC, ^#^ *p* < 0.05, ^##^ *p* < 0.01, ^####^ *p* < 0.0001; compared with CCl_4_ group, *p < 0.05, **p < 0.01, ** *p < 0.001, ****p < 0.0001, ns: not significant.

Throughout the treatment period, the body weight of mice in all groups exhibited an upward trend (Figure 5A). This suggests that both TCs and HLSP had minimal effect on the animals’ eating behavior at treatment doses. Hepatomegaly, as evidenced by the mean relative liver weight (also known as liver index), was induced by CCl_4_, with the CCl_4_ group having a significantly higher liver index than the NC group (*p* < 0.01). Treatment with TCs and HLSP, on the other hand, resulted in a significant decrease in liver index compared to the CCl_4_ group (*p* < 0.01), with no significant difference observed between the HLSP and MD groups (Figure 5B). The disturbance of the hepatocyte membrane structure caused by CCl_4_ led to a substantial rise in serum AST and ALT levels (*p* < 0.0001) (Figures 5C,D). Dose-dependent treatment with TCs resulted in a significant and substantial drop in ALT and AST levels in the MD and HD groups (*p* < 0.01 and *p* < 0.0001) (Figures 5C,D), with no significant difference observed between the HLSP and MD groups. It can be concluded that TCs can prevent hepatomegaly in CCl_4_-induced mice.

### 3.5 Histopathological observation

**FIGURE 6:**
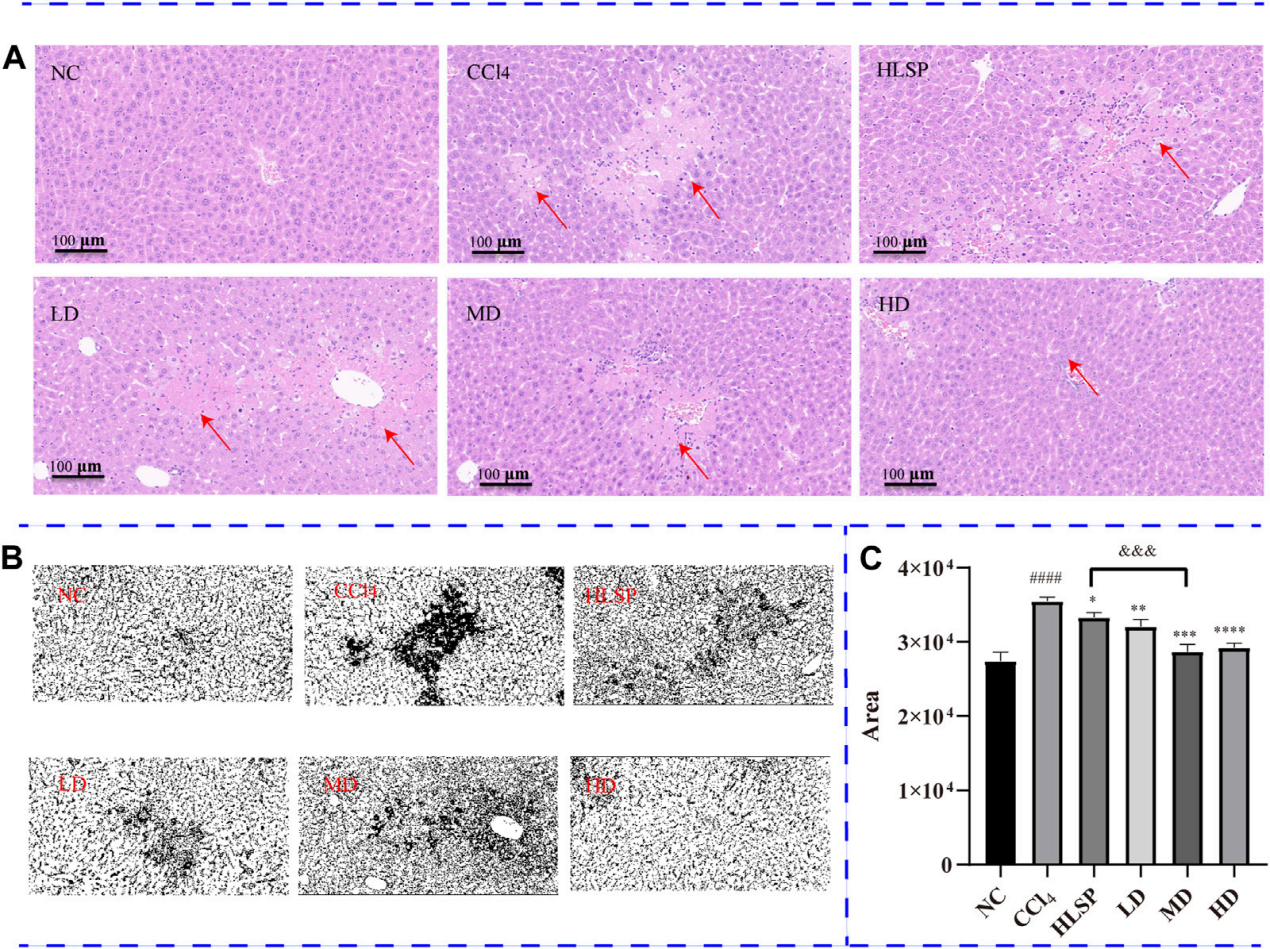
**(A)** H&E-stained liver sections of each group (HE×200), **(B)** RGB channels separation of the original HE staining image of liver tissue sections in each group. The figure shows the green stack under the appropriate threshold. The black part represents the degree of focal necrosis of liver. The darker the color, the greater the degree of necrosis. **(C)** Quantifying H&E-stained liver tissue area using ImageJ (x ± s, *n* = 3). Note: compared with NC group, ^#^ *p* < 0.05, ^####^ *p* < 0.0001; compared with CCl_4_ group <0.0001, *p < 0.05, **p < 0.01, ***p < 0.001, ****p < 0.0001; compared with HLSP group, ^andand&^ *p* < 0.001.

The purpose of histopathological observation is to reveal morphological alterations in liver cells, as well as inflammatory cell infiltration and other pathological conditions, which cannot be fully captured by serum biochemistry. Histological sections of liver tissue showed that the NC group had a morphologically normal liver lobular and cellular structure (Figure 6A). In contrast, the CCl_4_ group exhibited clear localised necrosis of hepatocytes, loss of hepatocyte structure, and infiltration of inflammatory cells, particularly around the central vein (indicated by the red arrow). Inflammatory cell infiltration was most common in the portal area and manifested as a decrease in nuclear volume, loss of cell structure, or aberrant cell shape. The HLSP group exhibited considerable improvement in hepatocyte shape and inflammatory infiltration. Perivascular inflammatory cell infiltration was lower in the LD group than in the CCl_4_ group. Increased dosages of TCs reduced inflammatory cell infiltration and improved cell morphology, resulting in a decrease in liver tissue necrosis area in a dose-dependent manner (Figures 6B,C). The HD group had the greatest hepatoprotective effect, reducing the necrotic area of liver tissue by 77.9% compared to the CCl_4_ group (*p* < 0.0001). The hepatoprotective effect showed a significant dose-response relationship, with the MD group performing marginally better than the HLSP group (*p* < 0.001), and the HD group performing the best.

### 3.6 Metabolite analysis and biomarker identification

**FIGURE 7:**
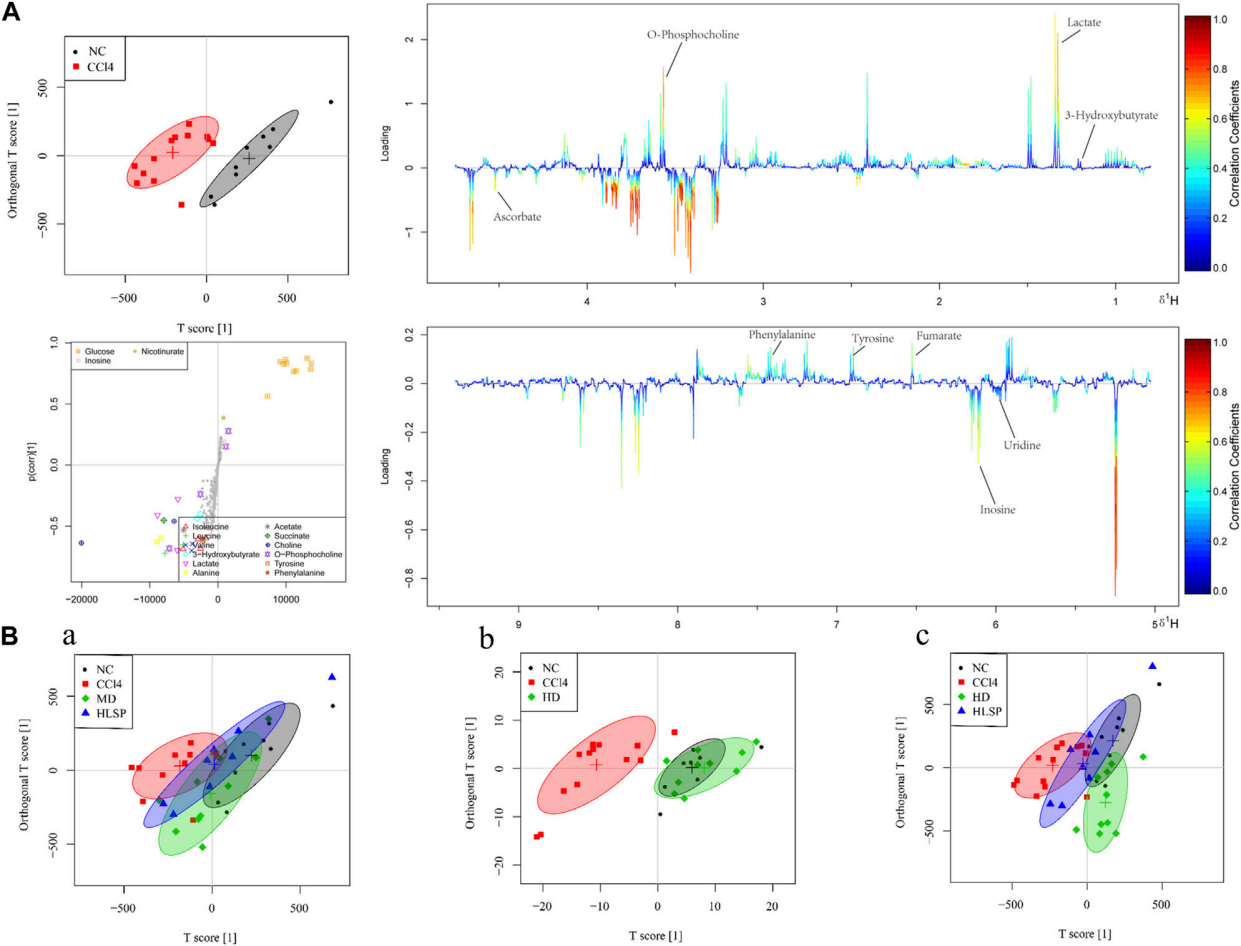
**(A)** OPLS-DA analysis of liver samples from mice in NC and CCl_4_ groups. Score plots (upper left), corresponding S plots (bottom left), and color-coded loading plots (right). The change from blue to red represents a significant increase in change. **(B)** OPLS-DA score plot of NC, CCl_4_, HLSP, MD and HD.

**FIGURE 8:**
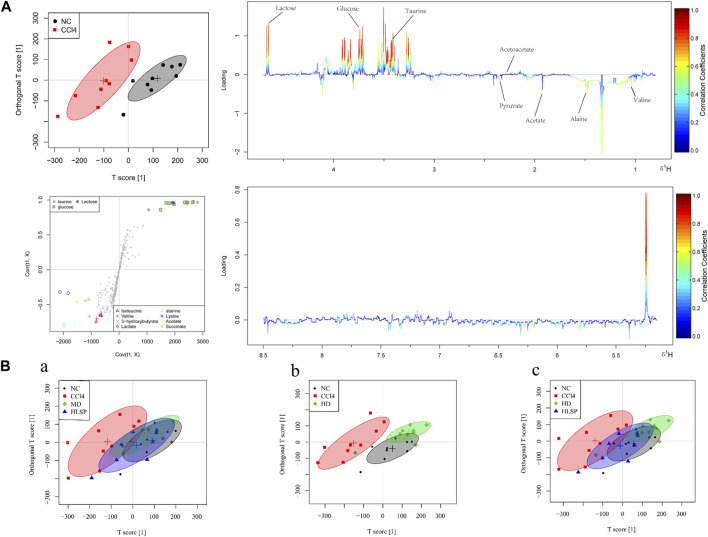
**(A)** OPLS-DA analysis of serum samples from mice in NC and CCl_4_ groups. Score plots (upper left), corresponding S plots (bottom left), and color-coded loading plots (right). The change from blue to red represents a significant increase in change. **(B)** OPLS-DA score plot of NC, CCl_4_, HLSP, MD and HD.

The main metabolic differences across groups were investigated using the OPLS-DA model, and the CCl_4_ and NC groups were clearly separated in liver and serum samples (Figure 7A; Figure 8A). Following TCs therapy, both the MD and HD groups distinguished themselves from the CCl_4_ group (Figures 7Ba and 8Ba), and the HD group nearly overlapped with the NC group (Figures 7Bb and 8Bb). The HD group exhibited greater separation from the CCl_4_ group than the HLSP group (Figures 7Bc and 8Bc), suggesting that TCs can dose-dependently reverse CCl_4_-induced metabolic abnormalities in mice.

**FIGURE 9:**
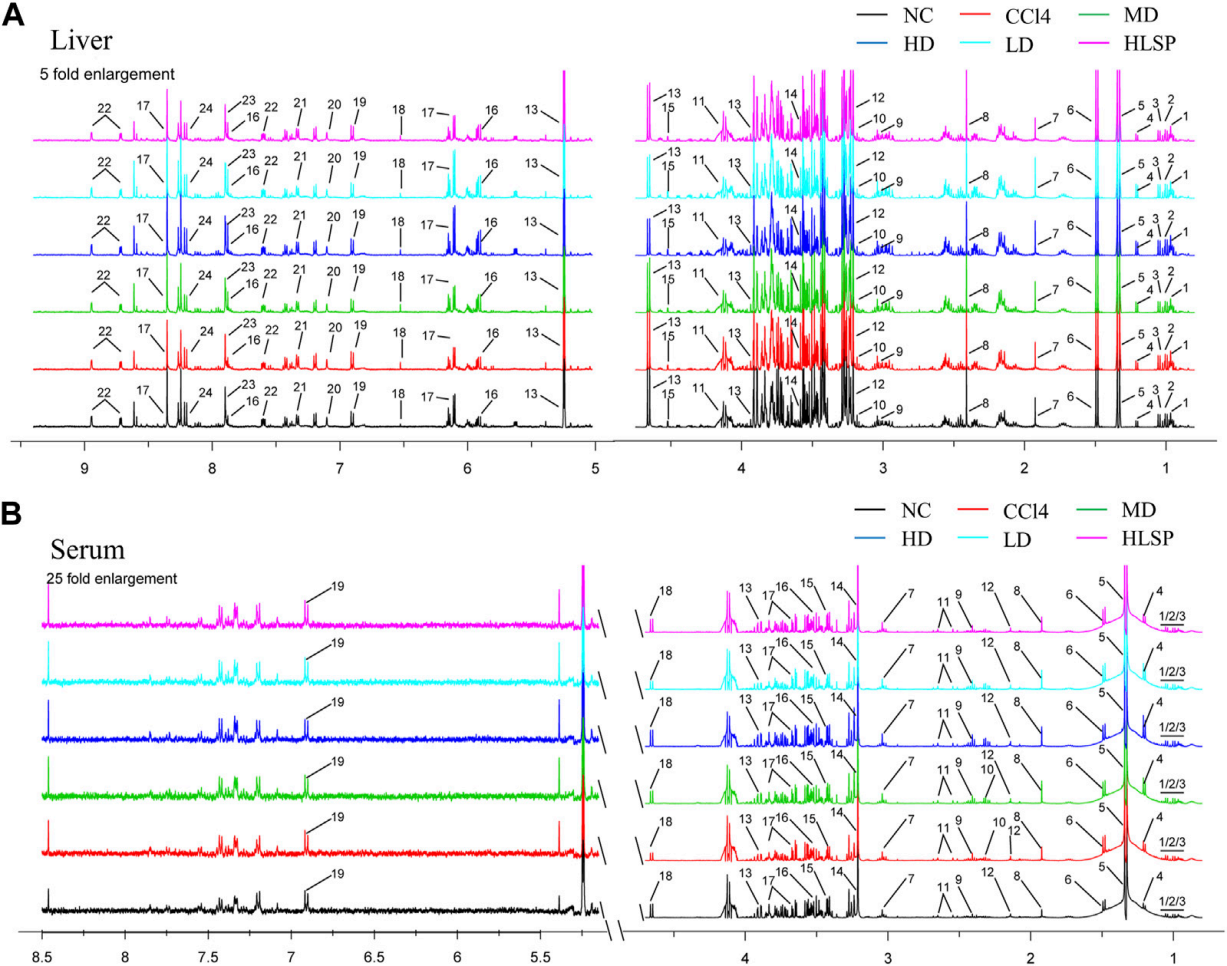
Typical 500 MHz ^1^H NMR spectra of liver and serum extracts. **(A)** Typical 500 MHz hydrogen spectrum of liver extract, 1, Isoleucine; 2, Leucine; 3, Valine; 4, 3-Hydroxybutyrate; 5, Lactate; 6, Alanine; 7, Acetate; 8, Succinate; 9, Creatine; 10, Choline; 11, O-Phosphocholine; 12, Betaine; 13, Glucose; 14, Glycine; 15, Ascorbate; 16, Uridine; 17, Inosine; 18, Fumarate; 19, Tyrosine; 20, Histamine; 21, Phenylalanine; 22, Nicotinurate; 23, Xanthine; 24, Hypoxanthine. **(B)** Typical 500 MHz hydrogen spectrum of serum extract, 1, Isoleucine; 2, Leucine; 3, Valine; 4, 3-hydroxybutyrate; 5, Lactate; 6, alanine; 7, Lysine; 8, Acetate; 9, Succinate; 10, Pyruvate; 11, Citrate; 12, Acetoacetate; 13, creatine; 14, Choline; 15, taurine; 16, Glycine; 17, glucose; 18, Lactose; 19, Tyrosine.

Using the OPLS-DA model, different metabolites were represented by various-sized points of different colors and shapes in the S-plot (Figure 7 bottom left). Among these metabolites, a total of 19 endogenous metabolites were identified (Figure 9) in liver and serum, 11 metabolites were chosen for potential biomarkers to distinguish between the NC and CCl_4_ groups. Lactate, fumaric acid, succinic acid, inosine, O-phosphocholine, ascorbate, taurine, tyrosine, phenylalanine, valine, and alanine were chosen based on fold changes and associated *p*-values between groups (see Supplementary Tables S3, S4 for details).

## 4 Discussion

We qualitatively analyzed the chemical composition of TCs and found that it primarily consists of CuB, IsocuB, CuE and cucurbitacin derivatives by MN. The MN provided comprehensive compound profiling of the constituents in TCs and HLSP. Notably, we discovered for the first time that HLSP, a positive medication used for treating hepatitis and primary liver cancer, also contains IsocuB in addition to the previously reported CuB and CuE. This is attributed to our choice of acetonitrile rather than methanol as the mobile phase. After optimization, we found that isocratic elution with 50% acetonitrile can well separate CuB and its configuration isomer IsocuB, and achieve the resolution required for quantification. The three major components in TCs and HLSP showed an enormous variance in values of IsocuB and CuE. Interestingly, it confirmed our previous hypothesis that different species in the same plant family may lead to huge differences in chemical composition. The content of CuB in TCs was much higher than in HLSP, while the contents of IsocuB and CuE were much lower than in HLSP.

The plant species compared are affected by differences in their growth environments, which impacts gene expression and leads to variations in chemical profiles (Zhou et al., 2016; Nikalje et al., 2023). *H. pedunculosum* grows at high altitudes in cold and drought Tibet, while *C. melo* grows in warmer, humid lowland areas. The cold and drought environment upregulates genes related to cucurbitacin synthesis in *H. pedunculosum*, resulting in higher levels of CuB and its isomer compared to *C. melo*. This finding is consistent with literature showing cucurbitacin accumulation increases under drought stress (Mkhize et al., 2023). Ultimately, growing in different climates impacts the plant biochemistry and results in divergence of therapeutic efficacy and safety between taxa (Panossian, 2023), warranting further evaluation of understudied species like *H. pedunculosum* that may have unexplored benefits.

In safety evaluations, the LD_50_ value of TCs was 36.21 mg/kg BW, 2.6-fold more than the LD_50_ value of HLSP in mice, indicating its higher safety. Bartalis et al. found that the IC_50_ of CuE, CuB are 0.1 μM, 0.8 μM, respectively in HeLa cells (Bartalis and Halaweish, 2011). Therefore, we have a hypothesis that the lower content of CuE (3.6%) may be one of the factors leading to lower cell toxicity than HLSP. To our knowledge, no value of NOAEL as an important indicator of non-clinical experimental research has been published for HLSP. In this study, through continuous observation for 2 weeks of any clinical signs in mice treated with TCs (15 mg/kg BW), no deaths, abnormal behavior or other adverse effects were observed. Previous studies have reported that CuB at an oral dose of 1 mg/kg BW could effectively improve the abnormal liver function in mice induced by concanavalin A (Yang et al., 2020) and mitigated sepsis-induced pulmonary pathological damage in rats (Hua et al., 2017). In this study, TCs at a dose of 0.4 mg/kg BW lower than the above study could significantly reduce serum ALT and AST levels after CCl_4_-induced liver injury. In addition, the effective dosage of TCs were 37-fold and 90-fold less than LD_50_ and NOAEL values, respectively, which ensure the safety of TCs use in preclinical study.

Our study further explored the protective mechanism of TCs on CCl_4_-induced liver damage in mice through liver tissue and serum metabolomics. After CCl_4_ treatment, lactate involved in pyruvate metabolism and fumarate involved in the TCA cycle were significantly increased compared with the normal group, which disrupted the energy metabolism. Previous studies have been reported that increased levels of lactate and fumarate are markers of live injury (Wolf et al., 2017; Haonon et al., 2021; Ma et al., 2023). Whereas, TCs treatment restored the increased levels of lactate and fumarate to normal levels. This demonstrate that TCs treatment effectively regulates the imbalance of energy metabolism caused by liver injury. In addition, our study identified significant alterations associated with oxidative stress, and amino acid metabolism. Ascorbic acid and taurine have been reported to act as ROS free radical scavengers (Das et al., 2008; Jong et al., 2012; Tian et al., 2021). In this study, ascorbic acid and taurine in the liver and serum were significantly decreased, indicating that oxidative free radicals attacked and impaired the liver’s cell membrane. Liver cells store ascorbic acid and taurine to combat oxidative stress, but membrane damage allows their leakage from cells. This results in lower intracellular concentrations. However, after TCs treatment, ascorbic acid in liver tissue and taurine in serum were significantly increased. It demonstrated that TCs can decrease oxidative stress and protect cell membranes from oxidative damage. This result is consistent with a previous study that CuB and CuE minimised cell damage caused by oxidative stress under drought-stressed environments (Mkhize et al., 2023). Since the liver is the primary site of amino acid metabolism (Ammar et al., 2022), CCl_4_ injury leads to the disruption of amino acid metabolism. Increased phenylalanine levels have been associated to immunological activation and inflammatory reactions *in vivo* (Moosmann and Behl, 2000). In our study, CCl_4_-induced liver damage is commonly accompanied by an increased level of phenylalanine, indicative of activated inflammatory response.

Previous studies have reported that HPS and its major lignan components have hepatoprotective effects (Shen et al., 2015; Shen et al., 2016; Feng et al., 2018). However, the hepatoprotective effects of HPP have been seldomly investigated. This study provides new insights into the hepatoprotective effects of TCs from HPP. Although the results confirmed that TCs showed significant protective effects against carbon tetrachloride-induced liver injury in mice, and exhibited superior efficacy and safety compared to the positive control drug, further work is still needed, including studies in other experimental animals and human validation. Future research directions should investigate the synergistic effects of the three monomeric components in TCs, and their dose-toxicity and dose-efficacy relationships. Overall, by developing a hepatoprotective formulation from the HPP that is rich in bioactive cucurbitacins, this study aims to remedy both the wastage of resources through disposal of this byproduct as well as mitigate ecological damage. Characterizing the pharmacological potential of cucurbitacins in the underutilized pericarp facilitates full utilization of the plant which previously led to resource wastage and environmental pollution upon disposal. Repurposing this byproduct for medicinal applications therefore represents an important example of sustainable phytomedicine development with significant reference value.

## 5 Conclusion

Our study demonstrated that the chemical composition of TCs primarily consists of CuB, IsocuB, and CuE. Interestingly, we discovered for the first time that HLSP, a positive control medication used for treating hepatitis and primary liver cancer, also contains IsocuB in addition to the previously reported CuB and CuE. This suggests the possibility that HPP could serve as a raw material for HLSP. We established the NOAEL values of TCs for the first time and found that the LD_50_ of TCs was significantly higher than that of HLSP, indicating greater safety. TCs exhibit a greater capacity to alleviate CCl_4_-induced liver damage in mice relative to HLSP. In conclusion, our findings demonstrate the protective effect of TCs against CCl_4_-induced liver injury in mice and reveal their potential for development into a hepatoprotective drug. The chemical composition and safety evaluation provide a basis for quality control. These results lay a foundation for the production and application of HPP.

## Data Availability

The datasets presented in this study can be found in online repositories. The names of the repository/repositories and accession number(s) can be found in the article/Supplementary Material.
