# Supplementary material for: Total cucurbitacins from Herpetospermum pedunculosum pericarp do better than Hu-lu-su-pian (HLSP) in its safety and hepatoprotective efficacy
Source: Front Pharmacol. 2024 Feb 22;15:1344983. doi: 10.3389/fphar.2024.1344983 (PMC10919163; doi:10.3389/fphar.2024.1344983)
Supplement: Supplementary file 1 [file DataSheet1.ZIP › S1 and S2.docx]

Supplemental information


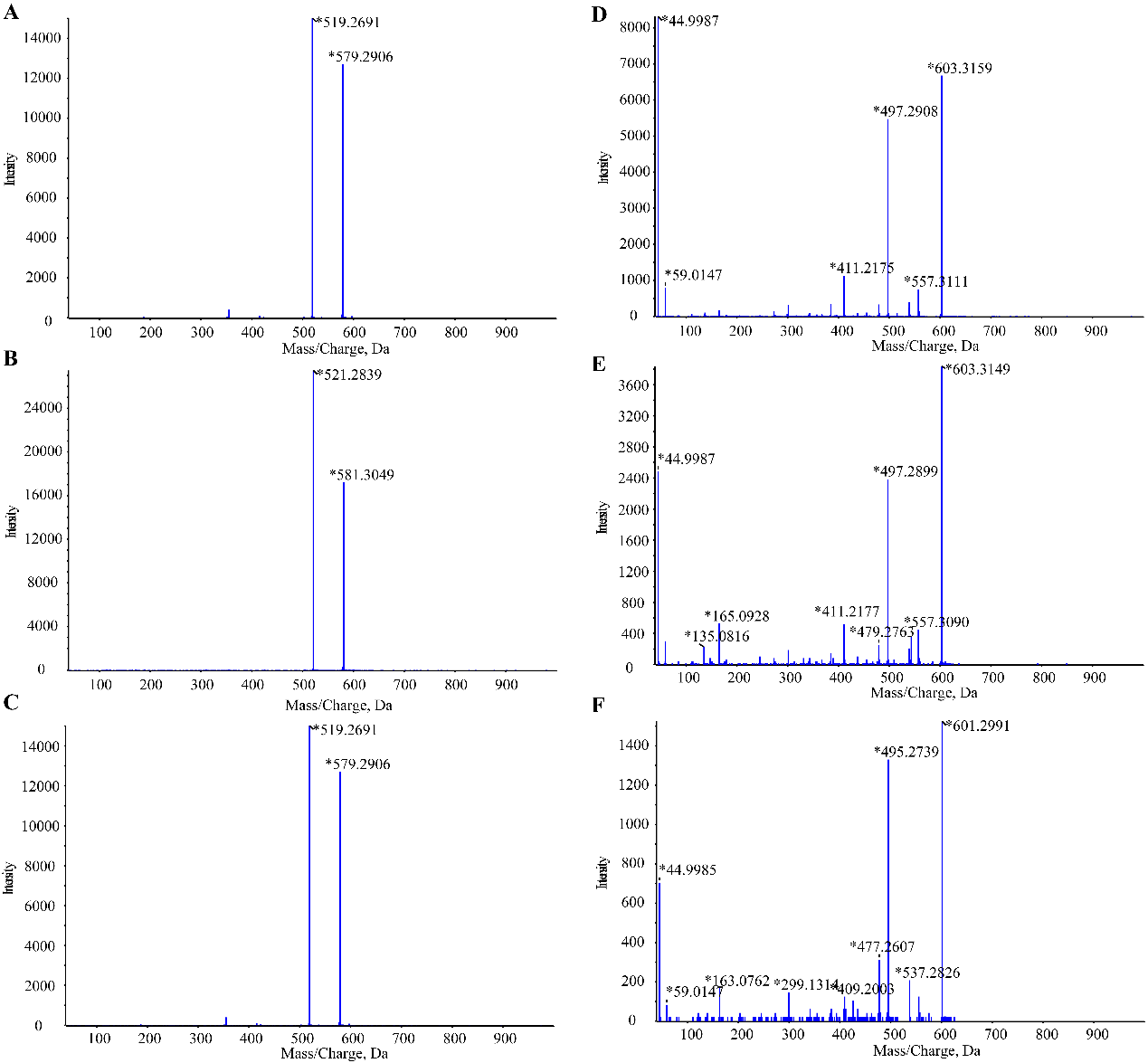


Figure S1 MS/MS spectra of Cucurbitacin B (A); Isocucurbitacin B (B); Cucurbitacin E (C) in ESI positive mode in TCs. MS/MS spectra of Cucurbitacin B (D); Isocucurbitacin B (E); Cucurbitacin E (F) in ESI negative mode in TCs.


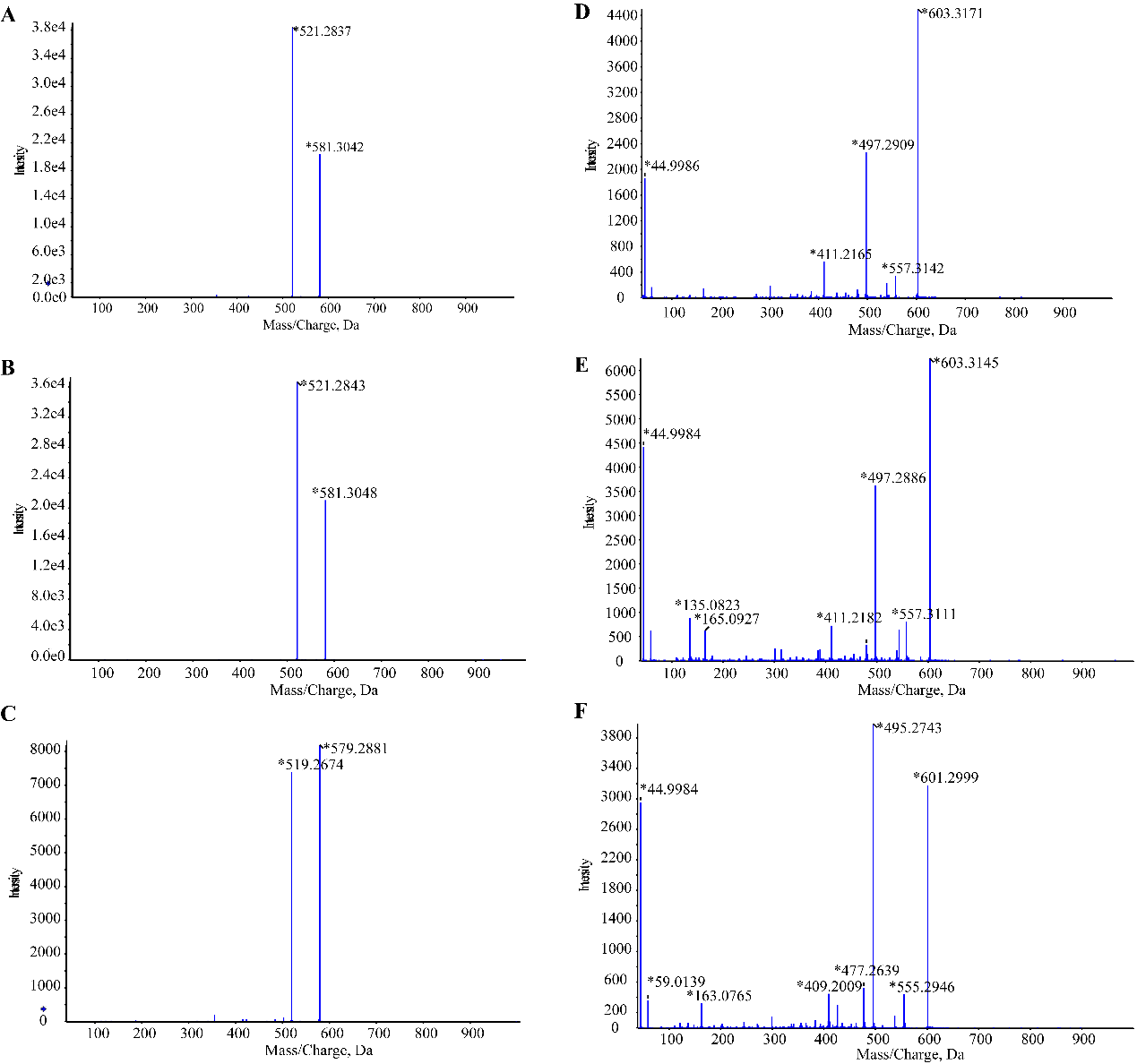


Figure S2 MS/MS spectra of Cucurbitacin B (A); Isocucurbitacin B (B); Cucurbitacin E (C) in ESI positive mode in HLSP. MS/MS spectra of Cucurbitacin B (D); Isocucurbitacin B (E); Cucurbitacin E (F) in ESI negative mode in HLSP.
